# Decoding a Rare Case of Pleomorphic Adenoma: A Case Report and Review of the Literature

**DOI:** 10.7759/cureus.68301

**Published:** 2024-08-31

**Authors:** Madhura Mahajan, Manjushri Waingade, Shreya Raghuwanshi, Anish Khatri, Rutuja N Mukkanwar

**Affiliations:** 1 Oral Medicine and Radiology, School of Dental Sciences, Krishna Vishwa Vidyapeeth, Karad, IND; 2 Oral Medicine and Radiology, Sinhgad Dental College and Hospital, Maharashtra University of Health Sciences (MUHS), Pune, IND; 3 Oral and Maxillofacial Surgery, SMBT Institute of Dental Sciences and Research, Nashik, IND; 4 Oral Pathology and Microbiology, Sinhgad Dental College and Hospital, Maharashtra University of Health Sciences (MUHS), Pune, IND

**Keywords:** surgical excision, benign tumor of palate, minor salivary gland tumor, palatal swelling, pleomorphic adenoma

## Abstract

Diagnosing palatal swellings is crucial for various reasons, mainly because these swellings can signal a range of health problems, from benign conditions to more serious diseases. Here, we have reported an interesting case of long-standing palatal swelling. Accurate diagnosis typically involves a combination of clinical examination, patient history, imaging studies, and possibly biopsy or other laboratory tests. Since each condition has unique characteristics and treatment approaches, differential diagnosis is essential for ensuring effective management. This case highlights the importance of considering pleomorphic adenoma in the differential diagnosis of palatal tumors and demonstrates the effectiveness of surgical management. A literature review is also presented, discussing the clinical, radiological, and histopathological features of this rare entity. Accurate diagnosis and timely intervention are crucial for effective treatment and maintaining overall oral health.

## Introduction

Palatal swelling refers to any abnormal growth or lesion that extends beyond the normal contours of the oral mucosa on the palate. Palatal swellings are clinically important as they may signal a spectrum of conditions, ranging from minor problems to more serious health issues [1-3]. Palatal lesions occur more frequently in elderly patients, and therefore, they have substantial clinical importance [3]. Differential diagnoses for palatal swellings include benign tumors (such as nasopalatine duct cyst), developmental anomalies (such as palatal tori), malignant tumors (such as squamous cell carcinoma and salivary gland tumors), infections (including candidiasis, mucormycosis, and abscesses), traumatic (such as hematomas and lacerations), inflammatory conditions (such as sarcoidosis and Behçet's disease), and systemic conditions (such as hematologic disorders). Precise diagnosis and prompt intervention are critical for effective treatment and the upkeep of overall oral health. This article aims to emphasize that while pleomorphic adenomas in the parotid salivary gland are the most commonly documented entities, one should consider pleomorphic adenoma as one of the significant differential diagnoses of palatal swellings.

## Case presentation

A 52-year-old female patient reported to the Department of Oral Medicine and Radiology with a chief complaint of swelling in the posterior palatal region. The swelling started five years ago; initially, it was smaller in size and gradually increased to the present size. No pain, paraesthesia, bleeding, or pus discharge was associated with the swelling. The patient had no contributory medical or family history and no relevant personal history of tissue abuse habits. She had undergone tooth extractions with upper and lower anterior and posterior tooth-bearing areas two years ago. The dental practitioner who did the dental extractions had advised her to undergo radiographic investigations for the palatal swelling, but she refused to do so as it was asymptomatic.

**Figure 1:**
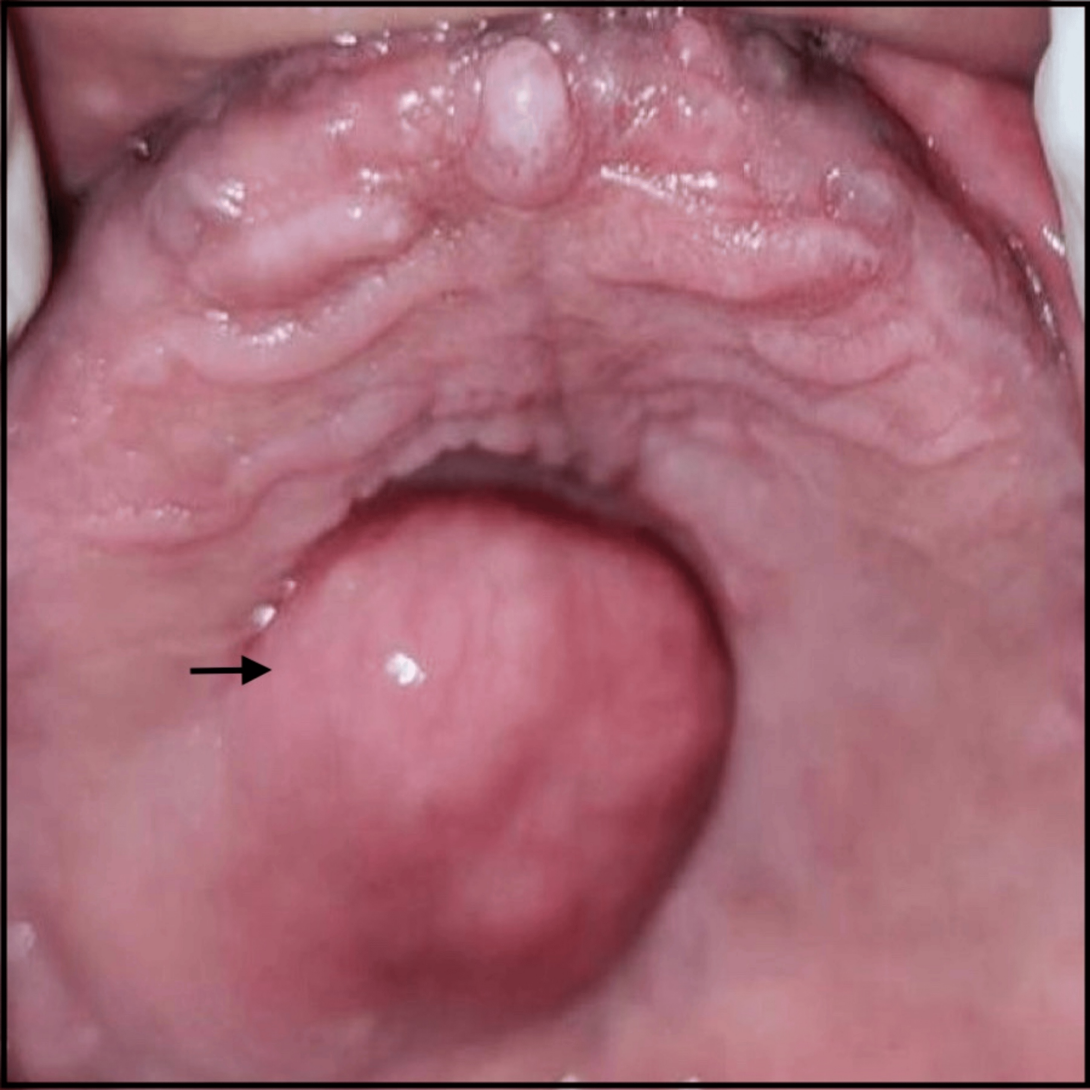
An intraoral solitary swelling (black arrow) on the posterior palatal surface and a completely edentulous maxillary arch.

Extraoral examination revealed an apparently symmetrical face, competent lips, adequate mouth opening, and non-palpable lymph nodes. Intraoral examination showed completely edentulous maxillary and mandibular arches. There was a well-defined, solitary, oval swelling present on the posterior palatal surface, which was extending anteroposteriorly approximately from 1.5 cm behind the incisive papilla to the junction of the hard and soft palate and mediolaterally from 0.5 cm beyond the mid palatine raphae to maxillary tuberosity (approximately 3 x 2 cm in size) (Figure 1). It had a sessile, soft to firm consistency and was non-tender on palpation.

**Figure 2:**
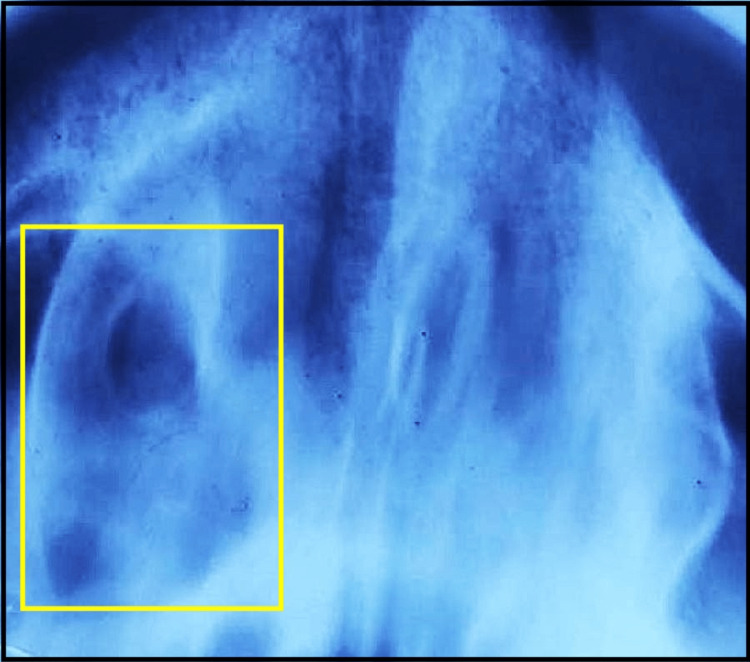
Maxillary occlusal radiograph showing palatal bone resorption (yellow box).

Based on the patient's history and clinical examination, a provisional diagnosis of salivary gland tumor with palate was given. Epulis, pleomorphic adenoma, maxillary sinus tumor, and mucoepidermoid carcinoma were considered as differential diagnoses. A maxillary occlusal radiograph showed palatal bone resorption (Figure 2).

**Figure 3:**
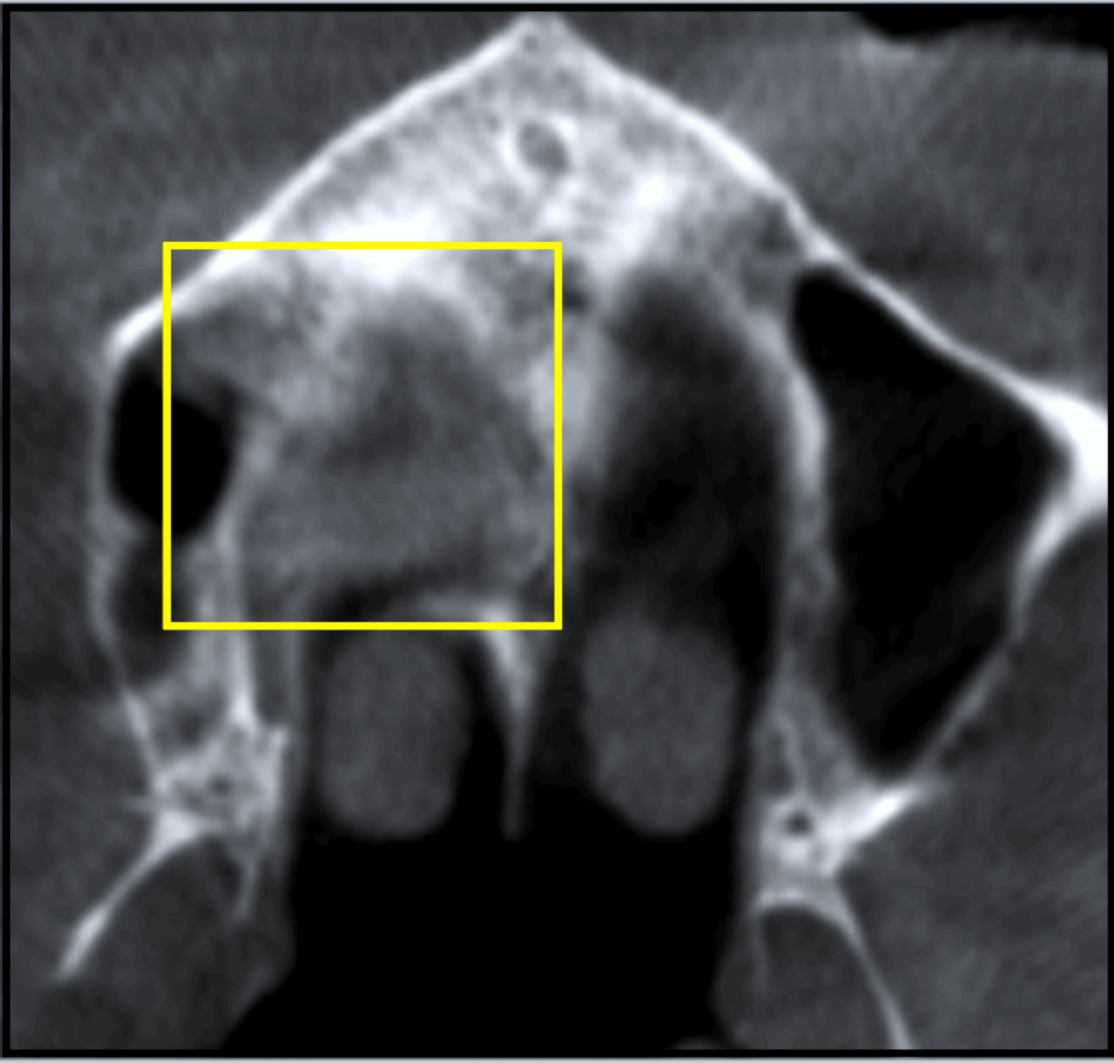
The axial section of the CBCT maxilla showing palatal bone resorption (yellow box) without cortical bone resorption. CBCT: cone-beam computed tomography

A cone-beam computed tomography (CBCT) of the maxilla revealed palatal resorption (Figure 3). The absence of cortical perforation was confirmed on axial, sagittal, and coronal sections.

**Figure 4:**
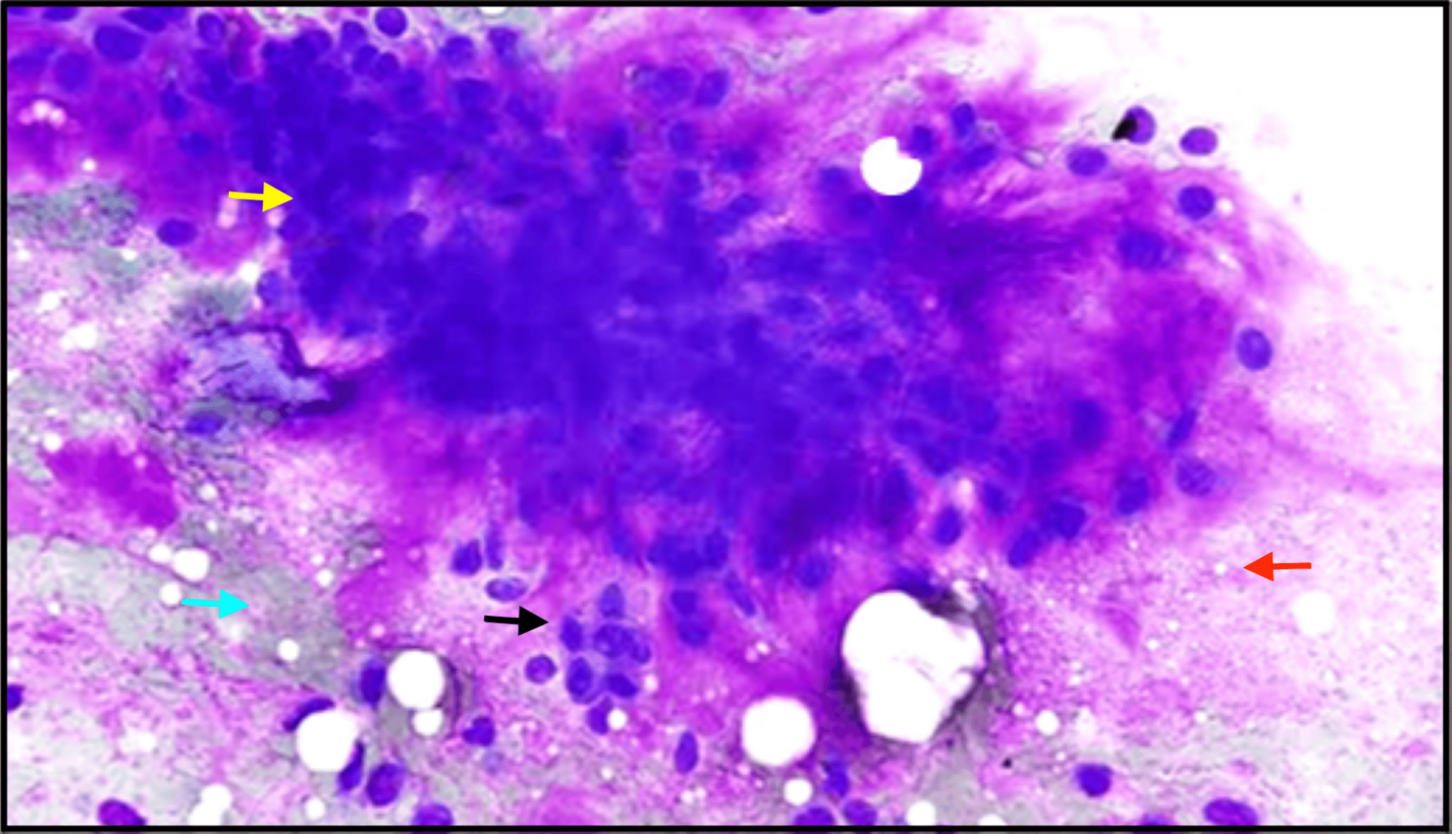
FNAC showed epithelial cells with pleomorphism (yellow arrow), cohesive clusters of ductal cells (red arrow), fragments of chondro-myxoid stroma (blue arrow), and myoepithelial cells (black arrow). FNAC: fine needle aspiration cytology

A fine needle aspiration cytology (FNAC) test was performed, which showed cohesive clusters of ductal cells and fragments of myxoid stroma with a fine fibrillar structure (Figure 4).

**Figure 5:**
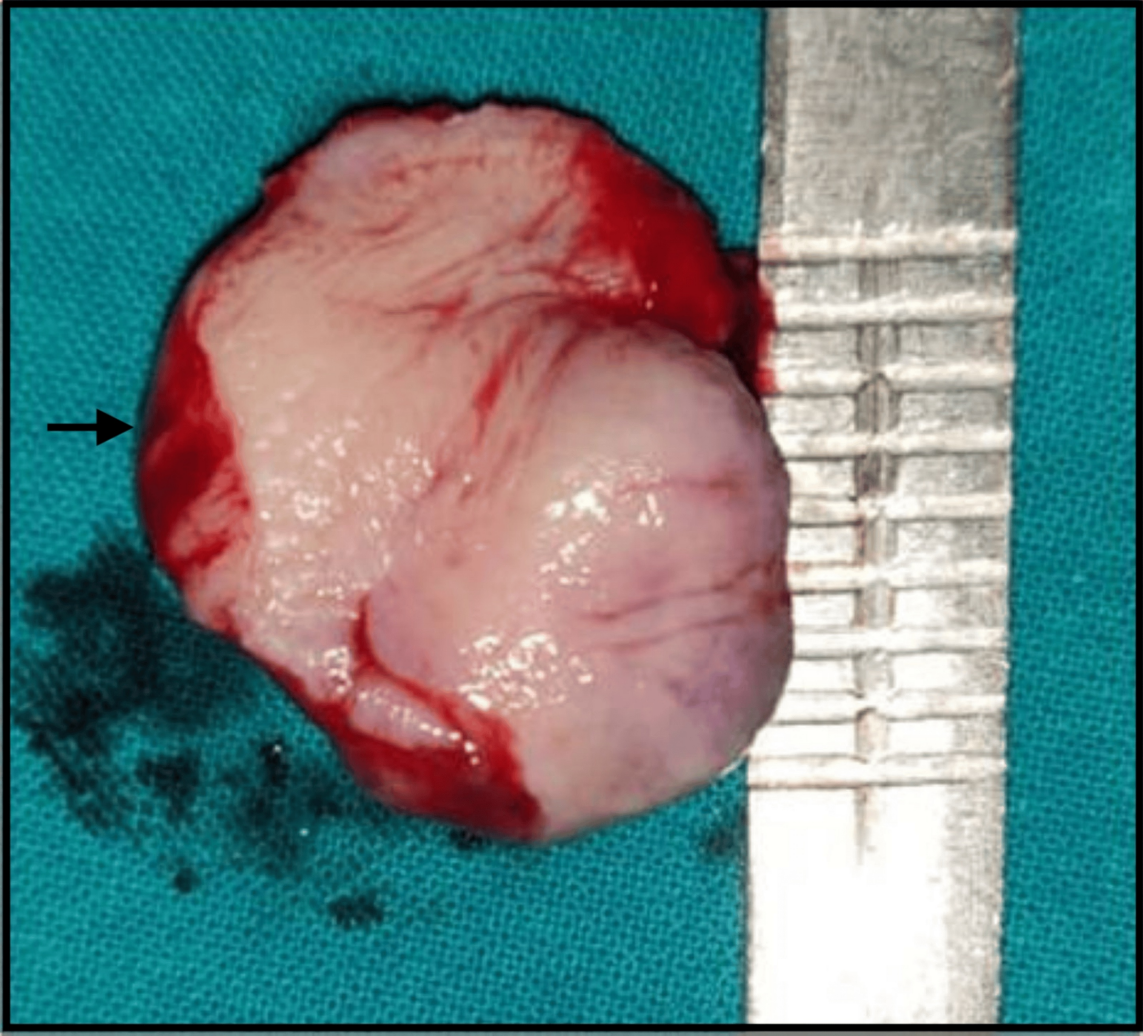
The excised specimen (black arrow) showed an encapsulated mass.

**Figure 6:**
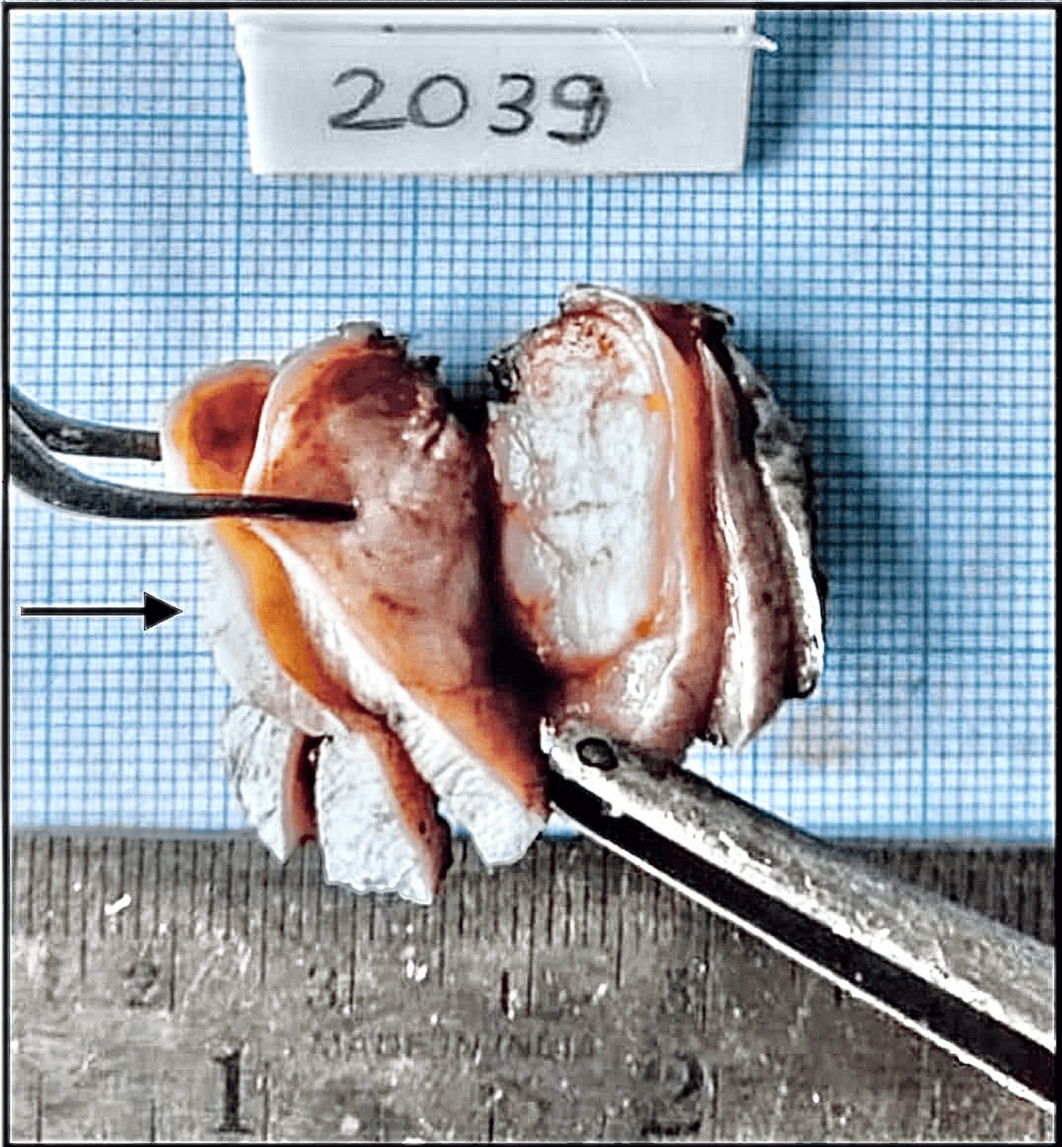
Excised specimen slicing (black arrow) taken for histopathological evaluation.

Excisional biopsy of the lesion, including the overlying mucosa with a 1 cm margin around the edges, was performed under local anesthesia and standard infection control procedures, followed by suture placement. The excised specimen showed an encapsulated mass measuring approximately 3.5 x 2.5 cm (Figures 5, 6).

**Figure 7:**
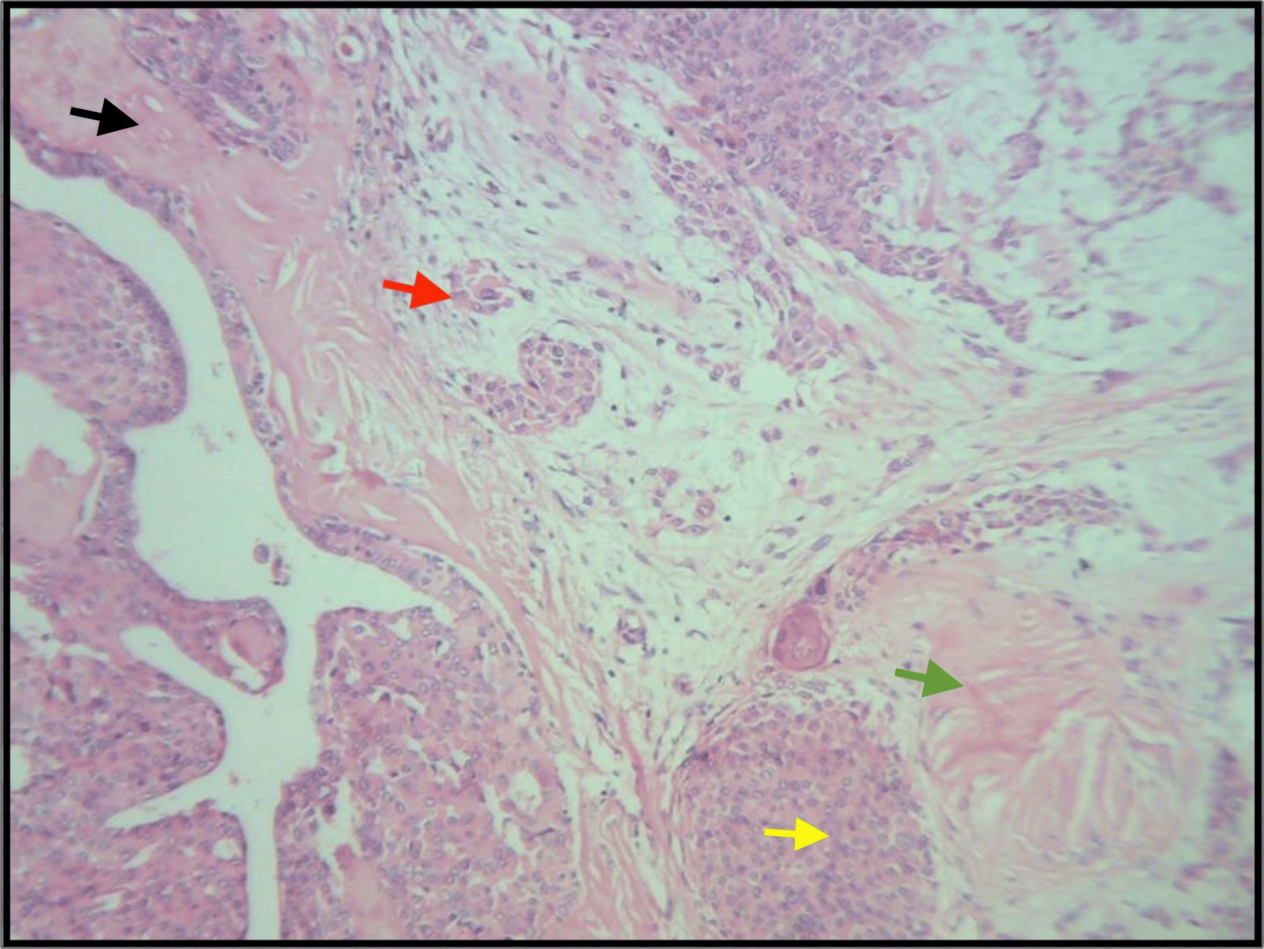
The H&E-stained ductal cells (yellow arrow), hyalinization (black arrow), and myoepithelial cells (red arrow), along with keratin pearl formation (green arrow), under 10× magnification. H&E: hematoxylin and eosin

**Figure 8:**
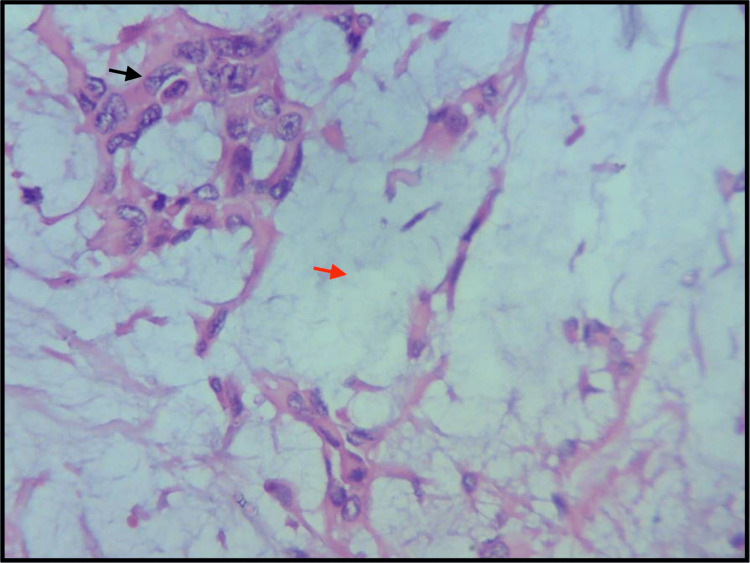
The H&E-stained ductal cells, myoepithelial cells (black arrow), and chondromyxoid tissue (red arrow) under 100× magnification. H&E: hematoxylin and eosin

The histopathological evaluation showed ductal cells and hyalinization, along with keratin pearl formation (under 10× magnification) (Figure 7) and ductal cells and myxoid tissue (under 100× magnification) (Figure 8).

The histopathological report was given as a pleomorphic adenoma. Given the potential for tumor recurrence, the patient will undergo routine check-ups every 12 months for the next two years.

## Discussion

Pleomorphic adenoma is the most frequent salivary gland tumor, consisting of two-thirds of all salivary gland neoplasms. As the name suggests, it has a pleomorphic origin from epithelial and myoepithelial elements [3-5]. The tumor's morphological complexity arises from the potential of cells to differentiate into various tissue types, including fibrous, hyalinized, myxoid, chondroid, and osseous areas, due to either metaplasia or the direct effects of the tumor cells themselves. It accounts for 53%-77% of parotid tumors, 44%-68% of submandibular tumors, and 33%-43% of minor salivary gland tumors [6]. The hard and soft palates are the most common locations for pleomorphic adenoma of the minor salivary glands, with the upper lip being the next most frequent site [6,7].

The exact etiology of pleomorphic adenoma is obscure. Few studies have suggested an association of the tumor with simian virus 40 (SV 40), tobacco use, genetic predisposition, and exposure to chemicals [6-9]. Molecular studies and cytogenetics have postulated chromosomal aberrations of 8q12 and 12q15 [7-10].

The diagnosis of pleomorphic adenoma is based on the patient's history, physical examination, cytological analysis, and histopathological findings. The tumors typically present as smooth and painless and usually remain asymptomatic until they grow to a large size, at which point they may cause pressure-related symptoms [11]. These tumors are generally well-defined from adjacent tissues by a pseudocapsule, which forms due to compression and fibrosis of the surrounding parenchyma. Additionally, the presence of small protrusions, or pseudopodia, on the tumor can contribute to the likelihood of recurrence [12].

CT and MRI scans are the preferred imaging techniques for determining the tumor's location, size, and extent to nearby superficial and deep structures [11,12]. These tumors can also invade and erode nearby bone, leading to radiolucent mottling on X-rays of the maxilla.

Histopathological analysis shows a tumor made up of clusters of stellate and spindle-shaped cells scattered within a myxoid stroma. The tumor's pleomorphic characteristics are defined by an internal layer of epithelial cells and an external layer of myoepithelial cells, which are arranged in diverse patterns with varying amounts of stroma [2,4]. Possible variations in the tumor may include squamous metaplasia, calcification, cartilage-like tissue, oxyphilic cells, and, although rare, malignant transformation [11-14].

The management of pleomorphic adenoma of the hard palate typically involves surgical removal of the tumor along with a margin of healthy tissue. If the tumor has infiltrated the periosteum or bone, these structures should also be excised. Generally, these tumors do not recur after proper surgical removal [15]. Recurrences can occur due to incomplete removal of the tumor, seeding, cutting through microscopic extracapsular projections and leaving residual tumor, or capsule rupture leading to inadvertent seeding of tumor cells, which is more likely when dissection is performed close to the capsule [13-16].

## Conclusions

Pleomorphic adenoma of the palate is rare and usually affects adults. It typically appears as a slow-growing, painless submucosal mass on the hard palate. A definitive diagnosis requires histopathological examination. The preferred treatment involves performing a surgical excision with wide margins, allowing the wound to heal naturally, which typically results in excellent outcomes. Although recurrences are infrequent, occasional cases may be identified during long-term follow-up.
